# Microbial Responses to the Reduction of Chemical Fertilizers in the Rhizosphere Soil of Flue-Cured Tobacco

**DOI:** 10.3389/fbioe.2021.812316

**Published:** 2022-01-11

**Authors:** Min-Chong Shen, Yu-Zhen Zhang, Guo-Dong Bo, Bin Yang, Peng Wang, Zhi-Yong Ding, Zhao-Bao Wang, Jian-Ming Yang, Peng Zhang, Xiao-Long Yuan

**Affiliations:** ^1^ Tobacco Research Institute of Chinese Academy of Agricultural Sciences, Qingdao, China; ^2^ College of Life Sciences, Qingdao Agricultural University, Qingdao, China; ^3^ Shandong Qingdao Tobacco Co., Ltd., Qingdao, China

**Keywords:** reduction of chemical fertilizer, bacterial community, variation of bacterial community, sustainable agriculture, agricultural resource utilization

## Abstract

The overuse of chemical fertilizers has resulted in the degradation of the physicochemical properties and negative changes in the microbial profiles of agricultural soil. These changes have disequilibrated the balance in agricultural ecology, which has resulted in overloaded land with low fertility and planting obstacles. To protect the agricultural soil from the effects of unsustainable fertilization strategies, experiments of the reduction of nitrogen fertilization at 10, 20, and 30% were implemented. In this study, the bacterial responses to the reduction of nitrogen fertilizer were investigated. The bacterial communities of the fertilizer-reducing treatments (D10F, D20F, and D30F) were different from those of the control group (CK). The alpha diversity was significantly increased in D20F compared to that of the CK. The analysis of beta diversity revealed variation of the bacterial communities between fertilizer-reducing treatments and CK, when the clusters of D10F, D20F, and D30F were separated. Chemical fertilizers played dominant roles in changing the bacterial community of D20F. Meanwhile, pH, soil organic matter, and six enzymes (soil sucrase, catalase, polyphenol oxidase, urease, acid phosphatase, and nitrite reductase) were responsible for the variation of the bacterial communities in fertilizer-reducing treatments. Moreover, four of the top 20 genera (unidentified JG30-KF-AS9, JG30-KF-CM45, *Streptomyces*, and *Elsterales*) were considered as key bacteria, which contributed to the variation of bacterial communities between fertilizer-reducing treatments and CK. These findings provide a theoretical basis for a fertilizer-reducing strategy in sustainable agriculture, and potentially contribute to the utilization of agricultural resources through screening plant beneficial bacteria from native low-fertility soil.

## Introduction

Chemical fertilizers have resulted in the prosperity of agriculture worldwide (Jez et al., 2016). Unfortunately, because of the excessive use of chemical fertilizers, inappropriate fertilization strategies are responsible for the degradation of the physicochemical properties and microbial structure of agricultural soil (Sun et al., 2019). A healthy status of the agricultural soil, which is determined by a balanced microbiome, is vital to plants (Chen et al., 2021). The effects of the physicochemical environment on the functions of the microbiome are linked to the comprehensive structures of the microbial communities. With the overuse of chemical fertilizers, especially nitrogen fertilizer, comes the recruitment of less beneficial bacteria and more pathogens in rhizosphere soil of plants (Achary et al., 2017; Saad et al., 2020), which leads to an ecological issue against sustainable agriculture.

In the People’s Republic of China, the Ministry of Agriculture and Rural Affairs have proposed and started to request the reduction of the amounts of chemical fertilizers through its Policy I Document from 2015. Thus far, a series of experiments that evaluated the current methods for reducing the amounts of chemical fertilizers (Liu et al., 2021) and their effects on soils and plants were implemented on main food crops (rice, wheat, and maize) (Maltas et al., 2013; Geng et al., 2019). However, fewer aspects were researched in commercial crops than those in food crops. In China, tobacco is one of the most important commercial crops and has improved the income of farmers in tobacco-planting areas. To maintain the income, continuous planting and excessive chemical fertilizers are implemented in tobacco-planting soil (Anthony and Ferroni, 2012; Yang and Fang, 2015), which has deteriorated the biotic and abiotic properties of the soil. Regarding the requirements of tobacco for nitrogen, phosphorus, and potassium, phosphorus and potassium fertilizers are needed in larger amounts compared to those of other food crops (López-Arredondo et al., 2014; Heuer et al., 2017; Lisuma et al., 2020). Although the tobacco plant requires high levels of phosphorus- and potassium-containing fertilizers, its requirement for nitrogen is the highest. However, among the three major chemical fertilizers, the most serious damages to the soil environment are caused by nitrogen fertilizers. Therefore, reductions on nitrogen fertilization in the cultivation of tobacco can not only improve the economic benefits for tobacco farmers (Liu et al., 2020), but also help restore soil health and maintain the stability of the soil ecosystem.

In this study, a nitrogen-reducing strategy, which employs three different nitrogen-reducing levels (10, 20, and 30%), was implemented and the effects of these reduced levels on the composition and structure of the soil bacterial communities were investigated, based on diversity analyses. The correlation of the top 50 genera was demonstrated by heatmap analysis. The environmental factors that impacted the bacterial communities of the different treatments were studied. Furthermore, the biomarkers of the nitrogen-reducing strategies and the conventional fertilization (as the control group, CK) were analyzed. Subsequently, four of the top 20 genera were identified as key bacteria. These findings helped to understand the bacterial responses to the different gradient reductions of nitrogen fertilizer in tobacco-planted soil, which may provide a theoretical basis for screening beneficial bacteria from native habitats of soils with overuse of chemical fertilizers.

## Materials and Methods

### Design of Field Experiments

This experiment was conducted in the Huangdao District, Qingdao, Shandong Province (32°01′40.61″ N, 120°10′53.60″ E), China, from March 19 to October 23, 2019. The local area has a subtropical monsoon climate, with an average annual rainfall of 1,062.3 mm, an annual average temperature of 15.3°C, an annual sunshine duration of 2,114.6 h, an annual frost-free period of about 218 days, and an average relative humidity of 80%. The previous crop in the experiment site was tobacco (Zhongyan 100 variety), which has been planted for three consecutive years. The flue-cured tobacco variety used in this experiment was the Zhongyan 100. The basic fertilizers of different treatments were implemented at April 22 according to the fertilization strategies (Table 1). The tobacco was transplanted at May 1, and potassium nitrate was applied as additional fertilizer at June 3.

**TABLE 1:** Fertilization strategies of field experiments.

Treatments	Fertilization strategies
CK	Fermented soybeans (N:P:K = 6:1:2), 300 kg ha^−1^; Tobacco fomulated fertilizer (N:P:K = 10:10:20), 324 kg ha^−1^; Diammonium Phosphate (N:P:K = 18:46:0), 30 kg ha^−1^; Potassium sulfate (N:P:K = 0:0:50), 153 kg ha^−1^
D10F	Fermented soybeans (N:P:K = 6:1:2), 270 kg ha^−1^; Tobacco fomulated fertilizer (N:P:K = 10:10:20), 291.6 kg ha^−1^; Diammonium Phosphate (N:P:K = 18:46:0), 27 kg ha^−1^; Potassium sulfate (N:P:K = 0:0:50), 167.16 kg ha^−1^; Calcium Superphosphate (N:P:K = 0:20:0), 24.6 kg ha^−1^
D20F	Fermented soybeans (N:P:K = 6:1:2), 240 kg ha^−1^; Tobacco fomulated fertilizer (N:P:K = 10:10:20), 259.2 kg ha^−1^; Diammonium Phosphate (N:P:K = 18:46:0), 24 kg ha^−1^; Potassium sulfate (N:P:K = 0:0:50), 181.32 kg ha^−1^; Calcium Superphosphate (N:P:K = 0:20:0), 49.2 kg ha^−1^
D30F	Fermented soybeans (N:P:K = 6:1:2), 210 kg ha^−1^; Tobacco fomulated fertilizer (N:P:K = 10:10:20), 226.8 kg ha^−1^; Diammonium Phosphate (N:P:K = 18:46:0), 21 kg ha^−1^; Potassium sulfate (N:P:K = 0:0:50), 195.48 kg ha^−1^; Calcium Superphosphate (N:P:K = 0:20:0), 73.8 kg ha^−1^

### Collection and Preprocessing of Soil Samples

Physicochemical experiments: The soil samples were collected 60 days after tobacco transplantation. A soil extractor was used to collect bulk soil (0–20 cm) (Fonseca et al., 2018) avoiding the fertilizer sites during the harvest period. The soil sample was brought to the laboratory, evenly spread, and located in an undisturbed place away from direct sunlight to dry naturally. The air-dried soil was ground, passed through 60-mesh and 100-mesh sieves, placed in a Ziploc bag, and sealed for storage until physicochemical properties were tested.

DNA extraction: The samples of tobacco rhizosphere soil, the one that adheres closely to tobacco roots, was collected using a sterilized brush and gentle shaking (Pétriacq et al., 2017). Three replicates were conducted for each treatment. The soil samples were immediately stored in a refrigerator at −20°C for extraction of soil DNA.

### Determination of Soil Physicochemical Properties

The soil pH was measured using the potentiometric method, and the soil-liquid ratio was 1:2.5. The soil organic matter was determined through the oil bath heating potassium dichromate oxidation volumetric method (Chen et al., 2016). The soil moisture content was measured using the drying method (Su et al., 2014). The total nitrogen in the soil was determined through the Kjeldahl distillation method (Sáez-Plaza et al., 2013). The available phosphorus in the soil was leached with sodium bicarbonate-hydrochloric acid, and then determined using the molybdenum-antimony anti-colorimetric method (Han et al., 2020). The available potassium in the soil was first extracted with nitric acid, and then determined with the flame photometer method (Lu et al., 2017).

### Determination of Soil Enzymes

Soil invertase was determined using the 3,5-dinitrosalicylic acid colorimetric method (Dong et al., 2019). Catalase was measured through a volumetric method (Yang et al., 2015). The activity of polyphenol oxidase was determined through the pyrogallol colorimetric method (Goyeneche et al., 2013). Soil urease was determined with the indophenol colorimetric method (Jing et al., 2020). Soil phosphatase was determined with the disodium phenyl phosphate method (Shao et al., 2020). Soil nitrite reductase was first leached with sodium nitrite-aluminum potassium alum, and then measured through a colorimetric method (Safonov et al., 2018).

### DNA Extraction and Amplicon Sequencing of 16S rRNA Genes

Each 0.5 g soil sample was obtained from well-mixed rhizosphere soil from one random replication. DNA was extracted from the soil using a DNA extraction kit (FastDNA^**TM**^ SPIN Kit for soil, MP Biomedicals, LLC, Solon, OH, United States) according to the manufacturer’s instructions. Subsequently, the DNA was tested with 1% agarose gel and the successfully extracted DNA was stored at −20°C immediately.

The PCR of the V4-V5 variable region of the bacterial 16S rRNA genes was conducted using the specific primers 515F (5′-GTGCCAGCMGCCGCGGTAA-3′) and 907R (5′-CCG​TCA​ATT​CCT​TTG​AGT​TT-3′) (Fan et al., 2018). The amplification products were tested for specificity in 1% agarose gel. Then the library was constructed using the library construction kit TruSeq^®^ DNA PCR-Free Sample Preparation kit (Illumina, San Diego, CA, United States). After the library was successfully constructed, the quantitative process was implemented using the Qubit^®^ 2.0 Fluorometer (Life Technologies, Carlsbad, CA, United States) and qPCR. After the quantitative test, subsequent sequencing was performed on the Illumina MiSeq platform.

### Processing and Analyses of Bioinformatic and Soil Data

The raw data after sequencing was spliced trough FLASH (V1.2.11, https://ccb.jhu.edu/software/FLASH/index.shtml) to obtain raw tags. Then QIIME software (V1.9.1, http://qiime.org/scripts/split_libraries_fastq.html) was used to filter raw tags. Subsequently, the Usearch (Version 7.0, http://www.drive5.com/usearch/) was used to detect and remove chimeras and get effective data (effective tags) (Callahan et al., 2016).

The valid data from all samples were clustered into the same operational taxonomic units (OTUs), the most frequently occurring sequence was screened as the representative sequence of OTUs, and the rRNA database SILVA (V138, https://www.arb-silva.de/) for species annotation analysis was used (Singleton et al., 2021). The phylogenetic relationship of all OTUs was constructed with the MUSCLE software (Version 3.8.31, http://www.drive5.com/muscle/) (Lu et al., 2019). Finally, all OTUs were uniformized based on the sequencing data of the sample with the smallest amount of data as the standard.

Follow-up analysis was performed based on the OTU information after the normalization process. Qiime software (Version 1.9.1, http://qiime.org/install/index.html) was used to calculate Shannon, Simpson, Ace, Chao1, PD whole tree indexes, and the R software (Version 3.6.0) was used to draw a dilution curve. Subsequently, Qiime software (Version 1.9.1) was used to calculate the Bray-Curtis distance, and the WGCNA, stats, and ggplot2 packages of the R software (Version 3.6.0) were used to draw PCoA diagrams. The Vegan package based on the R software (Version 3.6.0) was used to test the differences in the microbial community structure among different treatments through PERMANOVA. The Wilcoxon rank sum test was used to calculate the significance *p*-value in the LEfSe analysis, and the LDA was set to 3.0. Based on the analysis platform of Majorbio (Shanghai Majorbio Bio-pharm Technology Co., Ltd., Shanghai, China), the results of species annotations were used for further comparative analyses. All sequence data were submitted to the Sequence Read Archive (accession number: PRJNA780371) and are freely available at the NCBI (https://www.ncbi.nlm.nih.gov/sra/PRJNA780371).

The soil physicochemical properties data were analyzed using SPSS (Version 25.0) and the single factor ANOVA (Duncan’s Multiple Range Test) in SPSS software was used to calculate the significance of the differences between different treatments. All pictures were drawn with Microsoft Excel (Version 2019), Origin (Version 2018) (OriginLab Corporation, Northampton, MA, United States) and the R software (Version 3.6.0). All results are presented as the mean ± standard deviation.

## Results

### Reduction of Nitrogen Fertilizer Impacted the Composition of the Bacterial Communities

**FIGURE 1:**
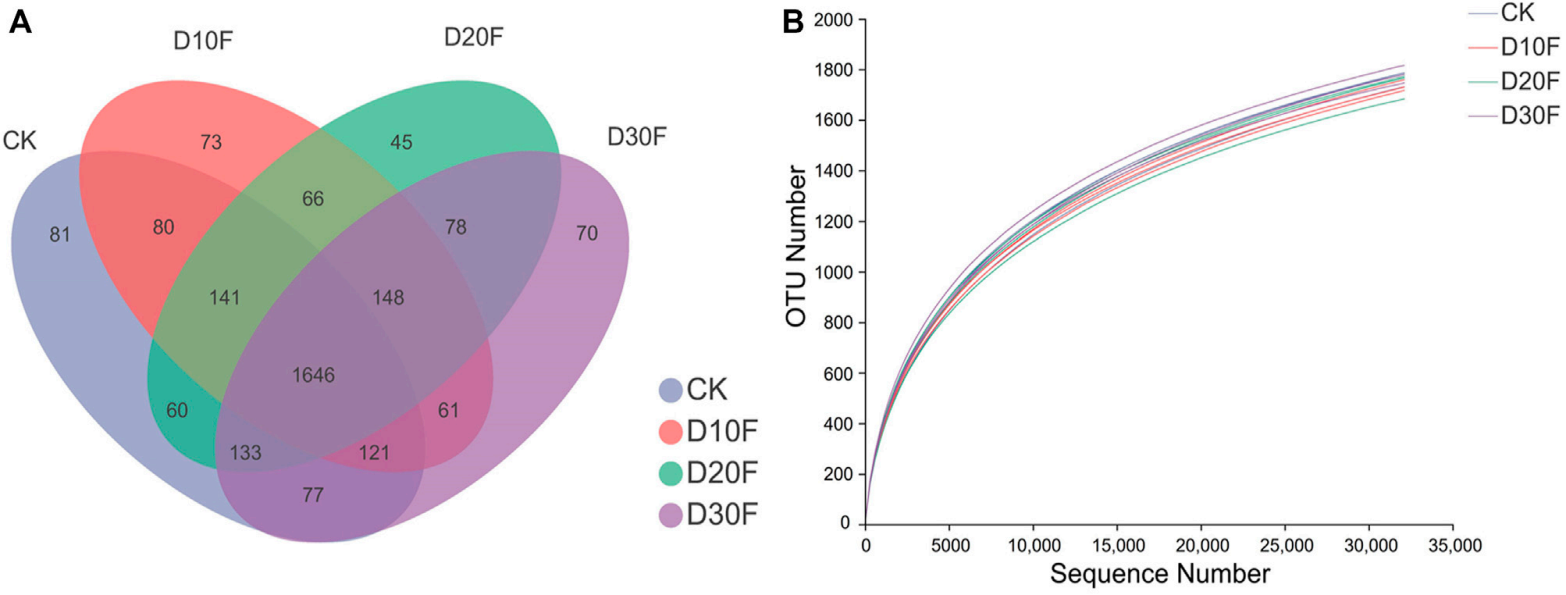
Comparison of the bacterial communities in different treatments based on OTU numbers. **(A)** Venn diagram of OTU numbers for different treatments. Every circle indicates a treatment; the number of OTUs shared between different treatments was interpreted with the number in the overlapping circles, while the number in the non-overlapping area represented the number of unique OTUs for the specific treatment. **(B)** Rarefaction curves of bacterial communities for all treatments.

The Venn diagram illustrated the dissimilarity of the effects of different reductions in the amount of nitrogen fertilizer on bacterial communities based on OTU numbers. There were 1,646 shared common OTUs in all treatments, whereas CK, D10F, D20F, and D30F presented 81, 73, 45, and 70 unique OTUs, respectively (Figure 1A). The rarefaction curve showed that the abundance of bacterial communities in CK was higher than that of the fertilizer-reducing treatments, consistently with the results of the Venn diagram (Figure 1B). Additionally, the Simpson index of the alpha diversity decreased in the fertilizer-reducing treatments. D20F significantly (*p* < 0.05) decreased the Simpson index, when compared to CK, which indicated that the diversity of the bacterial community declined in D20F whereas, there were no significant differences in the other indices of alpha diversity (Table 2).

**TABLE 2:** Statistic results of Alpha diversity indices of different treatments.

Sample name	Shannon	Simpson	Ace	Chao1	PD whole tree
CK	5.5763 ± 0.1364	0.0199 ± 0.0061a	2,261.33 ± 19.21	2,269.99 ± 44.07	130.38 ± 1.69
D10F	5.6680 ± 0.0668	0.0130 ± 0.0020ab	2,265.46 ± 25.63	2,260.42 ± 23.13	129.19 ± 0.68
D20F	5.6973 ± 0.1181	0.0116 ± 0.0022b	2,234.97 ± 64.75	2,217.07 ± 105.33	128.12 ± 3.98
D30F	5.7011 ± 0.1065	0.0147 ± 0.0029ab	2,233.57 ± 45.20	2,220.84 ± 69.04	131.81 ± 2.27

All data in the table are presented as means ± standard deviation (SD). Means followed by different lower-case letters are significantly different at the 5% level by DMRT (Duncan multiple range test).

**FIGURE 2:**
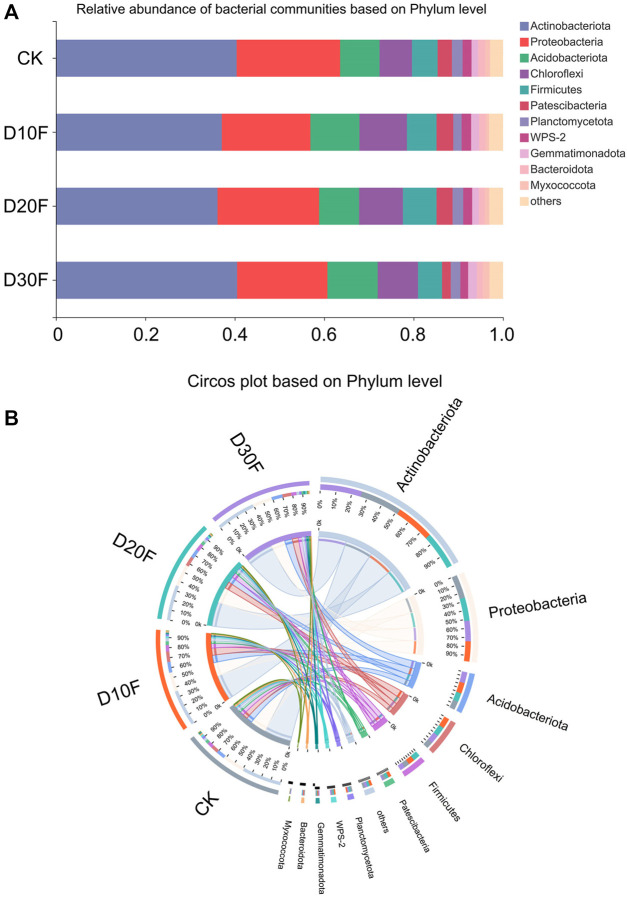
Composition of the bacterial communities for different treatments, based on phylum. **(A)** Bar plot of relative abundance of bacterial communities based on phylum. **(B)** Circos plot of different treatments based on phylum.

The taxonomic identification was implemented based on OTUs, and the top five dominant phyla were Actinobacteriota, Proteobacteria, Acidobacteriota, Chloroflexi, and Firmicutes (Figure 2A). Among them, the relative abundance of Actinobacteriota decreased in the D10F and D20F treatments, whereas it increased in D30F. The relative abundance of Proteobacteria decreased in the fertilizer-reducing treatments, whereas the relative abundance of Acidobacteriota and Chloroflexi increased in the treatments. In addition, D20F and D10F resulted in the largest relative abundance of Firmicutes and Patescibacteria, respectively, in all treatments. Meanwhile, D30F reduced the relative abundance of both Firmicutes and Patescibacteria, when compared to CK (Figure 2B). The top five dominant genera were identified as *Arthrobacter*, *Gaiellales*, unclassified *Intrasporangiaceae*, *Sphingomonas*, and *Nocardioides*, followed by *Bacillus*, unidentified JG30-KF-AS9, unclassified *Acidobacteriales*, and *Streptomyces*. Among them, *Arthrobacter* and unclassified *Intrasporangiaceae* were extremely close on the phylogenetic tree; while *Nocardioides* and *Streptomyces* were secondarily close (Supplementary Figure S1).

### Reduction of Nitrogen Fertilizer Impacted the Structure of Bacterial Communities

**FIGURE 3:**
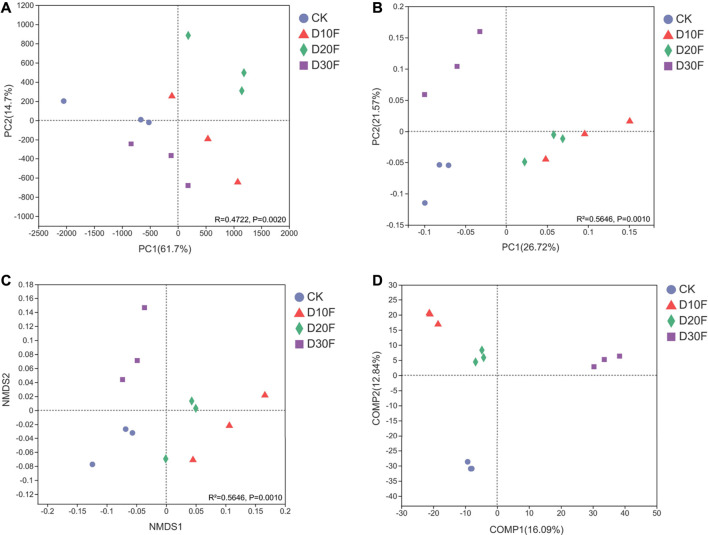
Combined plot integrated with the PCA **(A)**, PCoA **(B)**, NMDS **(C)**, and PLS-DA **(D)** plots of the bacterial communities corresponding to beta diversity.

The beta diversity for all treatments based on analyses of dimensionality reduction demonstrated the variation of bacterial communities between different treatments (Figure 3). The PCA plot (R = 0.4722, *p* = 0.0020) showed that the bacterial communities of different treatments were separated. Gaps between the bacterial communities for the D10F and D20F treatments were larger than those for D30F and CK (Figure 3A). Evident separation between the bacterial communities of the fertilizer-reducing treatments (D10F, D20F, and D30F) and CK was shown in the PCoA plot (*R* ^2^ = 0.5646, *p* = 0.0010), whereas the clusters for the D10F and D20F treatments did not show obvious dissimilarity between each other (Figure 3B). The results of the NMDS analysis (*R* ^2^ = 0.5646, *p* = 0.0010) were consistent with the PCoA, except that the partition of D10F and D20F occurred along the NMDS1 arrow (Figure 3C). To emphasize the differences between groups, PLS-DA was implemented with a supervised algorithm. The results indicated obvious variation of the bacterial communities among all treatments. Additionally, both bacterial communities of the D10F and D20F treatments were clustered in the same quadrant, while the clusters of the bacterial communities for D30F and CK were located in other two different quadrants (Figure 3D).

**FIGURE 4:**
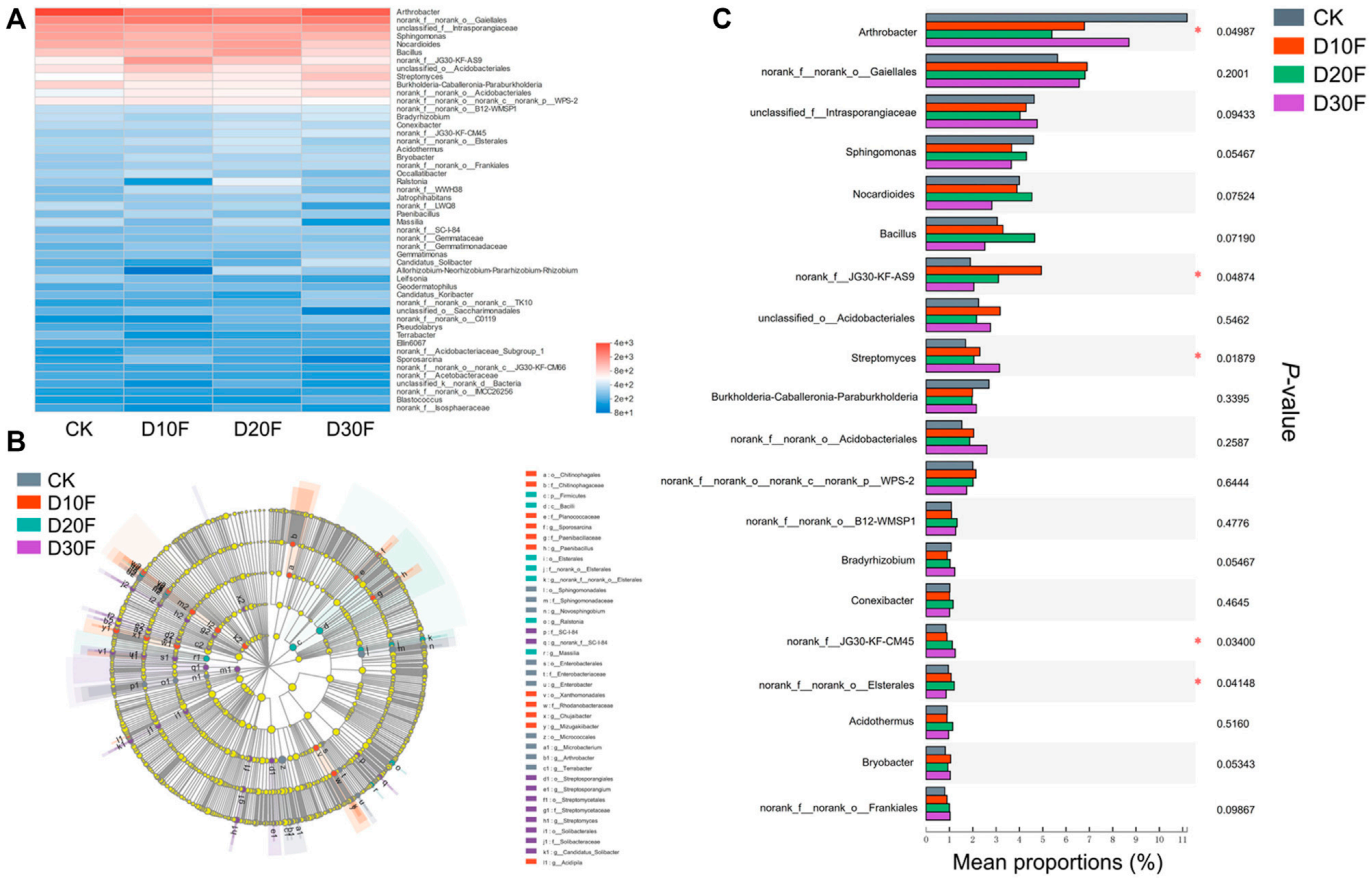
Variation of the bacterial communities in different treatments based on genus. **(A)** Heatmap of the top 50 genera based on logarithmic statistics of identified OTUs among different treatments. **(B)** LDA Effect Size (LEfSe) analysis based on the genus among different treatments. **(C)** Bar plot of relative abundance of the top 20 genera among different treatments based on the Kruskal-Wallis H test.

To further explore the structural differences of the bacterial communities among different treatments, investigations corresponding to differential genera were performed. The top 50 genera for all treatments were scanned and the heatmap was drawn based on their relative abundance. The result showed the differences in the bacterial composition between different treatments. The variation of the bacterial community composition between fertilizer-reducing treatments (D10F, D20F, and D30F) and CK was larger than that between D10F and D20F based on the genus, which was consistent with the results of PCoA, NMDS, and PLS-DA (Figure 4A). Furthermore, 76 taxa were analyzed as biomarkers of the corresponding treatments based on LEfSe analysis. Among them, 27 taxa were identified at the genus level, of which seven, eight, four, and eight were considered biomarkers for CK, D10F, D20F, and D30F, respectively (Figure 4B). Taking the relative abundance of each bacterial genus into consideration, the top 20 genera were investigated. The results indicated that four of the top 20 genera, unidentified JG30-KF-AS9, *Streptomyces*, unidentified JG30-KF-CM45, and *Elsterales*, were all significantly enriched in the fertilizer-reducing treatments, whose relative abundance increased in the D10F, D30F, D30F, and D20F, respectively. In addition, the relative abundance of *Arthrobacter* decreased in the fertilizer-reducing treatments (Figure 4C).

### Correlation Between Bacterial Key Taxa and Dominant Environmental Factors

**FIGURE 5:**
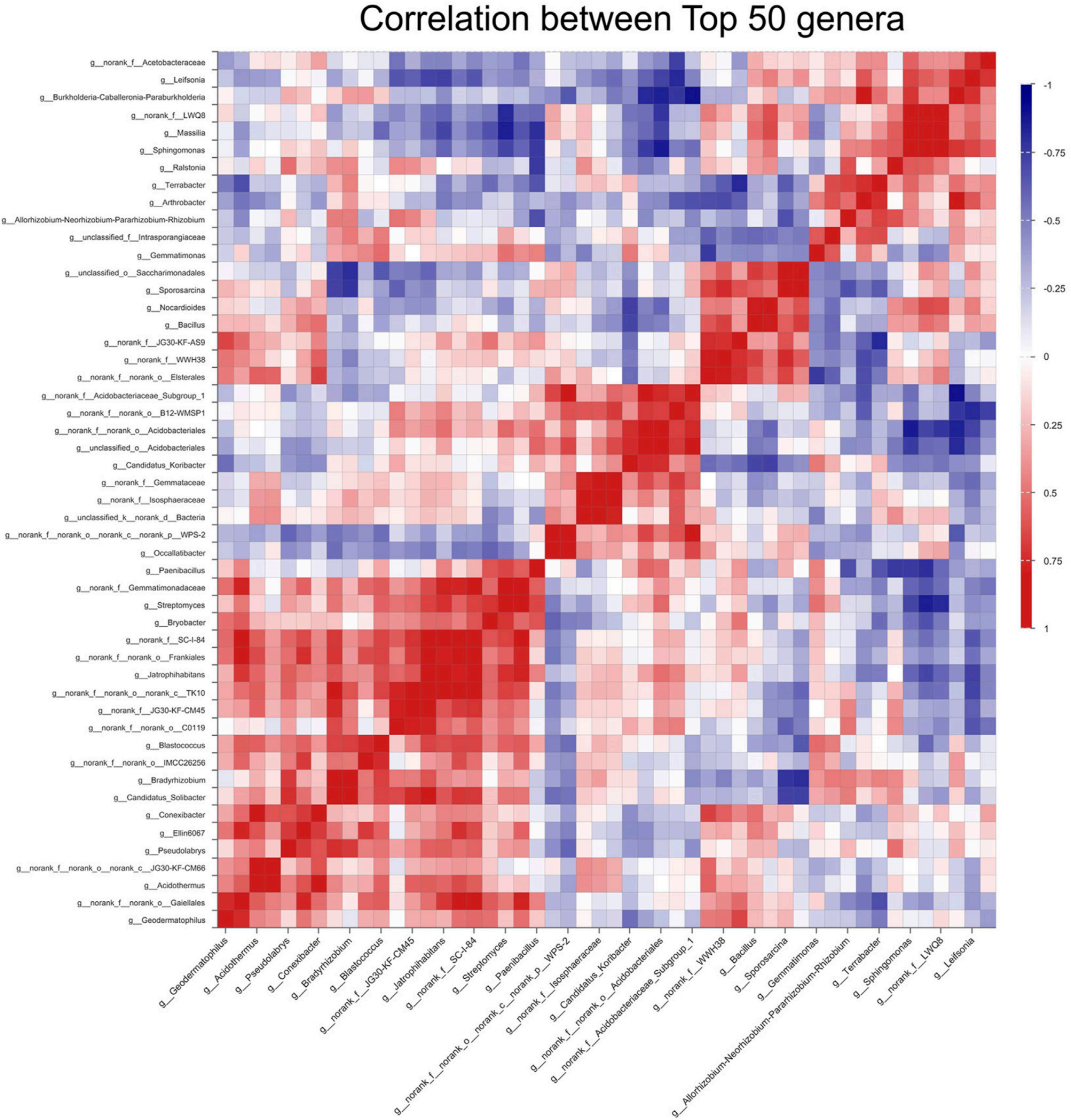
Heatmap of the correlation between Top 50 genera among different treatments.

To better understand the bacterial interaction and response to the reduction in the amount of nitrogen fertilizer, the correlation among the top 50 genera was explored through heatmap analysis (Figure 5) and statistical analysis based on OTUs (Supplementary Table S1). The results illustrated that unidentified JG30-KF-AS9, *Streptomyces*, unidentified JG30-KF-CM45, and *Elsterales* had 28, 27, 32, and 29 positive interactions, respectively, while they had 21, 22, 17, and 20 negative interactions, respectively. Among them, unidentified JG30-KF-AS9 had four significantly positive correlations to *Geodermatophilus*, *Elsterales*, unidentified WWH38, and *Sporosarcina*, and two significantly negative correlations to *Arthrobacter* and *Terrabacter*. *Streptomyces* had six significantly positive correlations to unidentified TK10, *Jatrophihabitans*, unidentified SC-I-84, *Bryobacter*, *Gemmatimonadaceae*, and *Paenibacillus*, and three significantly negative correlations to *Sphingomonas*, *Massilia*, and unidentified LWQ8. Unidentified JG30-KF-CM45 presented six significantly positive correlations to *Solibacter*, unidentified C0119, unidentified TK10, *Jatrophihabitans*, *Frankiales*, and unidentified SC-I-84, and one significantly negative correlation to *Leifsonia*. Additionally, *Elsterales* presented five significantly positive correlations to *Conexibacter*, unidentified WWH38, unidentified JG30-KF-AS9, *Bacillus*, and *Sporosarcina*, and three significantly negative correlations to *Gemmatimonas*, unclassified *Intrasporangiaceae*, and *Arthrobacter*.

**FIGURE 6:**
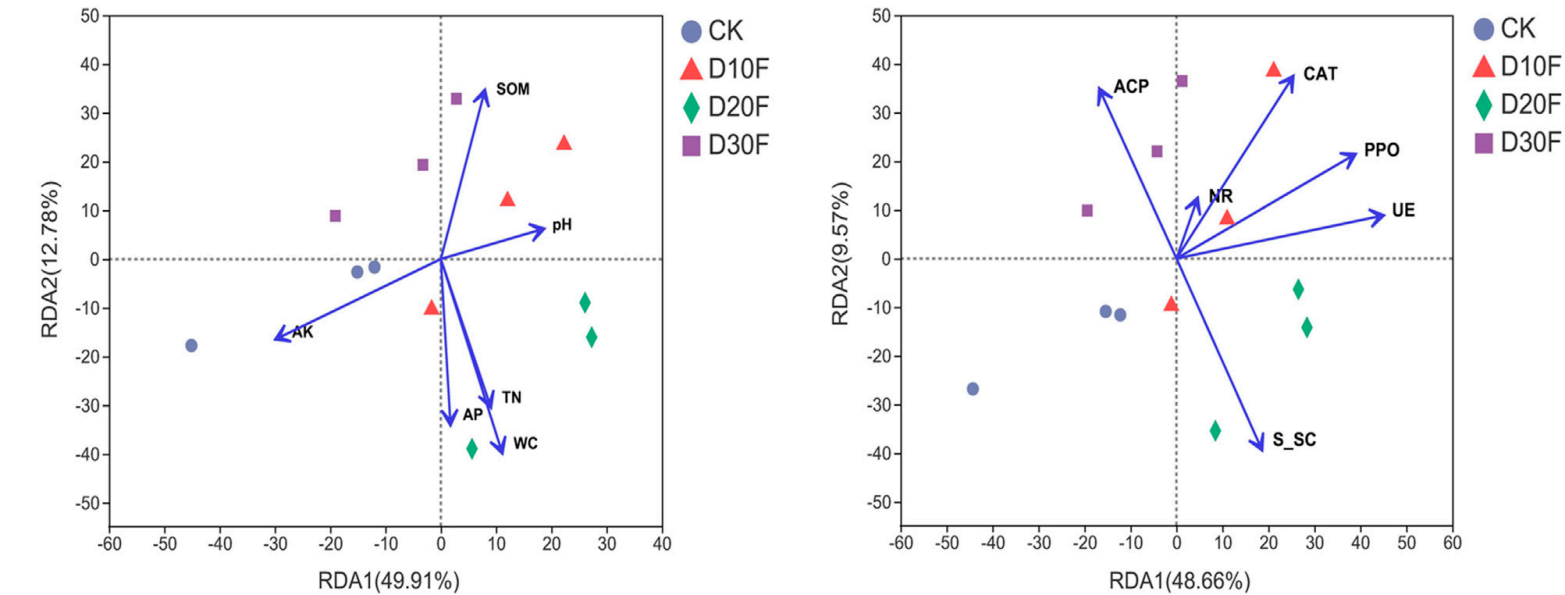
Redundancy analysis (RDA) of different treatments fitting the physicochemical indicators **(A)** and enzyme indicators **(B)** to the bacterial community data. The direction and length of the blue arrow lines indicate the explanation of different indicators, which influence the bacterial communities.

To further clarify the effect of environmental factors on the bacterial communities of tobacco-planting soil, redundancy analyses (RDA) of different treatments were implemented (Supplementary Tables S2, S3, Figure 6). The results of RDA showed that soil organic matter and pH drove the bacterial community structure in D30F and D10F treatments, respectively. Total nitrogen (TN), available phosphorus (AP), and water content (WC) dominated the bacterial community variation in D20F together, while available potassium (AK) drove the bacterial community of CK. In addition, RDA1 and RDA2 explained 49.91 and 12.78% of the bacterial community variation, respectively (Figure 6A). Regarding the effect of enzymes on bacterial communities, RDA1 and RDA2 explained 48.66 and 9.57% of the bacterial community variation, respectively (Figure 6B). Six enzymes were investigated and all the enzymes dominated the bacterial community variation of the nitrogen-reducing treatments. Among them, acid phosphatase (ACP) and soil sucrase (S_SC) dominated the bacterial community structure in the D30F and D20F treatments, respectively, whereas catalase (CAT), polyphenol oxidase (PPO), nitrite reductase (NR), and urease (UE) collectively drove the bacterial community variation in the D10F treatment. Additionally, the arrows of nitrite reductase and urease were pointing in the directions between D30F and D10F, and between D20F and D10F, respectively.

### Correlation Between Bacterial Key Taxa and Output Value of Tobacco Leaves

To verify the effects of nitrogen-reducing strategies on practical production of tobacco, the output value of tobacco leaves was evaluated based on the yield, ratio of first class, unit price and value indexes (Table 3). All nitrogen-reducing treatments could improve the ratio of first class and the unit price of tobacco leaves. Moreover, the yield of tobacco leaves was significantly (*p* < 0.05) increased by D10F and D20F, while the differences of yield between D10F and D20F, and CK and D30F were not significant. At the aspect of yield, D30F performed the worst in all treatments. Additionally, different unit prices, which were impacted by different treatments, brought significantly different output value of tobacco leaves. Among them, D10F made the highest output value, followed by D20F. Subsequently, D30F earned the lowest value, while there was no significant difference in output value between CK and D30F.

**TABLE 3:** Output value of tobacco leaves.

Treatments	Yield/kg ha^−1^	Ratio of first class/%	Unit price/RMB kg^−1^	Value/RMB ha^−1^
CK	2,242.35 ± 104.25a	60.34	24.80	55,611.52 ± 2,584.07a
D10F	2,555.85 ± 50.40b	63.71	25.96	66,348.57 ± 1,309.14c
D20F	2,448.75 ± 52.35b	62.12	25.35	62,075.82 ± 1,325.58b
D30F	2,167.65 ± 70.95a	61.24	25.04	54,276.70 ± 1774.91a

All data in the table are presented as means ± standard deviation (SD). Means followed by different lower-case letters are significantly different at the 5% level by DMRT (Duncan multiple range test).

Furthermore, the correlation between differential genera, referring to those were significantly different from CK based on OTU statistics, and output value of tobacco leaves were analyzed by mantel test (Table 4). The results demonstrated that the differential genera shared by D10F and D20F had positive correlations with yield and output value of tobacco leaves. Significant R value represented the key taxa made great contributions to tobacco yield and its economic value.

**TABLE 4:** Mantel test analysis of correlations between key taxa and output value of tobacco leaves.

	Key taxa (D10F-CF-D20F)	
	R Value	*p* Value
Yield	0.3533	0.027
Output Value	0.4419	0.005

Key taxa refer to those are significantly different from CK based on OTU statistics.

## Discussion

The excessive use of chemical fertilizers was proved to be one of the most fatal issues in agricultural production (Smith and Siciliano, 2015). To cope with the increasing population worldwide, sufficient, even overloaded chemical fertilizers were added to the agricultural soil. With the rapid improvement of the crop yields came the potential risk of environmental pollution due to macronutrients (nitrogen, phosphorus, and potassium), which resulted in disturbing the physicochemical properties of the agricultural soil (Hui et al., 2021). Planting obstacles corresponding to improper physicochemical properties of the soil had brought much confusion to the determination of responsibilities for the declined plant yield (Gurdeep and Reddy, 2015). An integrated effect of biotic and abiotic processes determines the capacity of ecological systems of the agricultural field (Tkacz and Poole, 2015; Freschet et al., 2021).

Previous studies had shown when the reduction in nitrogen fertilizer ranged from 10 to 30%, the fundamental crops including rice, wheat, and maize, performed better than with conventional fertilization (Migliorati et al., 2014; Hofmeier et al., 2015; Ding et al., 2018). This article focused on the effects of reducing nitrogen fertilization on the tobacco-planting soil. The alpha diversity of the bacterial community declined with the decrease in the amount of nitrogen fertilizer. The beta diversity of the bacterial community demonstrated that the reduction in the amount of nitrogen fertilizer differentiated the bacterial community of the rhizosphere soil from that under conventional fertilization. Moreover, the bacterial community varied according to the different reduction in the amount of fertilizer. Interestingly, the variation of the bacterial community increased since the nitrogen application rate was reduced by more than 30%, when compared to no more than 20% reduction of the fertilizer. Resulting from the decrease in the nutrient, more intense competition occurred within the bacterial community (Zhang et al., 2016). In the challenging process, the populations with poor competitiveness were outcompeted. Generally, the populations eliminated were located in limited and narrow ecological niches due to their low use efficiency of environmental resources and their imperfect competitive mechanism (Turnbull et al., 2013; Dini-Andreote et al., 2014). These weakly competitive bacteria were less potential to implement their functions. Hence, the surviving populations of the bacterial community after the strategy of reducing nitrogen fertilization were discussed.

With the changes in the diversity of the bacterial community, its functionality in soil ecological systems varied accordingly (Knelman et al., 2012; Eo and Park, 2016; Zhang et al., 2016). The bacterial genera whose relative abundance changed significantly were the key factors that leaded to the variation in the diversity of the bacterial community. Thorough analyses of the differential bacterial genera might help to clarify the direction and details of variation of the bacterial community, which reveals its subsequent potential changes of functions. Furthermore, the analyses can lead to the identification and screening of efficient functional strains based on the correlation analysis of the functional orientation of the bacterial community and key differential bacterial genera. Four genera, unidentified JG30-KF-AS9, JG30-KF-CM45, *Streptomyces*, and *Elsterales* were demonstrated as key species. In addition, all the environmental factors in our study were verified to dominate the variation of the bacterial community structures of the nitrogen-reducing treatments, except available potassium. The specific requirement of tobacco for potassium might have led to the changing direction of the bacterial community, which declined with the reduction in nitrogen fertilization.

Compared to conventional fertilization, the strategy of reducing nitrogen fertilization might change the relative abundance of JG30-KF-AS9 and JG30-KF-CM45 to improve the productive function of the bacterial community of the tobacco-planting soil (Chinta et al., 2021; Neupane et al., 2021; Zhang et al., 2021). Meanwhile, the reduction in nitrogen fertilizer might increase the relative abundance of *Streptomyces* (D30F) and *Elsterales* (D20F), which had the potential abilities to decrease the relative abundance of pathogens and their pathogenicity (Vurukonda et al., 2018; Wang et al., 2021; Momesso et al., 2022). The reduction in the amount of nitrogen fertilizer (10–30%) showed significant advantages for tobacco planting compared to CK, because of the soil microorganisms. Furthermore, this study found that JG30-KF-AS9, JG30-KF-CM45, *Streptomyces*, and *Elsterales* were beneficial genera that might potentially promote tobacco growth. Subsequent research on the isolation, enhancement, and application of these bacterial genera is of positive significance in the field of tobacco growth promotion and biological control.

Different from the strategies of adding exogenous microbial inoculants with plant-promoting or biological controlling functions, this research paid attention to the response of the native soil microbiome to the reduction of nitrogen fertilization without the addition of exogenous microorganisms. In this process, unidentified JG30-KF-AS9, JG30-KF-CM45, *Streptomyces*, and *Elsterales* demonstrated different functional vitalities from those when they were in the conventional habitat of CK. Native microorganisms have more potential to implement their functions due to stronger adaptability to the environment, when compared to exogenous microorganisms (Palozzi and Lindo, 2018). These findings have important guiding significance for screening indigenous highly functional microorganisms from poor soil environments, of which, the concept of turning waste into treasure is also conducive to the development of sustainable agriculture.

## Conclusions

In this study, the reduction in the amount of nitrogen fertilizer resulted in diverse responses of the soil bacterial community at the rhizosphere of the flue-cured tobacco plant. The structure of bacterial communities of tobacco-planting soil varied from decreasing 10–30% nitrogen fertilizer, and the variation of bacterial communities was enlarged, when compared to conventional fertilization, respectively. Furthermore, seven, eight, four, and eight genera were identified as biomarkers of CK, D10F, D20F, and D30F, respectively. Considering the relative abundance of all the OTUs, unidentified JG30-KF-AS9, *Streptomyces*, unidentified JG30-KF-CM45, and *Elsterales* were the key bacterial genera that caused structural and functional variations of soil bacterial communities among different treatments. Additionally, all macronutrients, except available potassium, along with pH, soil organic matter, and six enzymes dominated the variation of the bacterial communities in nitrogen-reducing treatments. This study provides a feasible strategy of reducing the amount of nitrogen fertilizer in the tobacco growing industry. Additionally, these results provide a theoretical basis for isolating functional bacteria from native microbial resources of relatively poor soil environment.

## Data Availability

The data presented in the study are deposited in the NCBI repository (https://www.ncbi.nlm.nih.gov/sra/PRJNA780371), accession number: PRJNA780371.
