# Supplementary material for: Microbial Responses to the Reduction of Chemical Fertilizers in the Rhizosphere Soil of Flue-Cured Tobacco
Source: Front Bioeng Biotechnol. 2022 Jan 11;9:812316. doi: 10.3389/fbioe.2021.812316 (PMC8787768; doi:10.3389/fbioe.2021.812316)
Supplement: Supplementary file 2 [file DataSheet1.docx]

***Supplementary Materials***

**Microbial Responses to the Reduction of Chemical Fertilizers in the Rhizosphere Soil of Flue-Cured Tobacco**

**Min-Chong Shen^1†^, Yu-Zhen Zhang^2†^, Guo-Dong Bo^1^, Bin Yang^3^, Peng Wang^3^, Zhi-Yong Ding^3^, Zhao-Bao Wang^2*^, Jian-Ming Yang^2^, Peng Zhang^1*^, and Xiao-Long Yuan^1*^**

^1^ Tobacco Research Institute of Chinese Academy of Agricultural Sciences, Qingdao 266101, China

^2^ College of Life Sciences, Qingdao Agricultural University, Qingdao 266109, China

^3^ Shandong Qingdao Tobacco Co., Ltd., Qingdao 266071, China

*** Correspondence:**Xiao-Long Yuan
yuanxiaolong@caas.cn

Peng Zhang
zhangpeng@caas.cn

Zhao-Bao Wang
wangzhaobao123@126.com

**Keywords: reduction of chemical fertilizer; bacterial community; variation of bacterial community; sustainable agriculture; agricultural resource utilization**

**
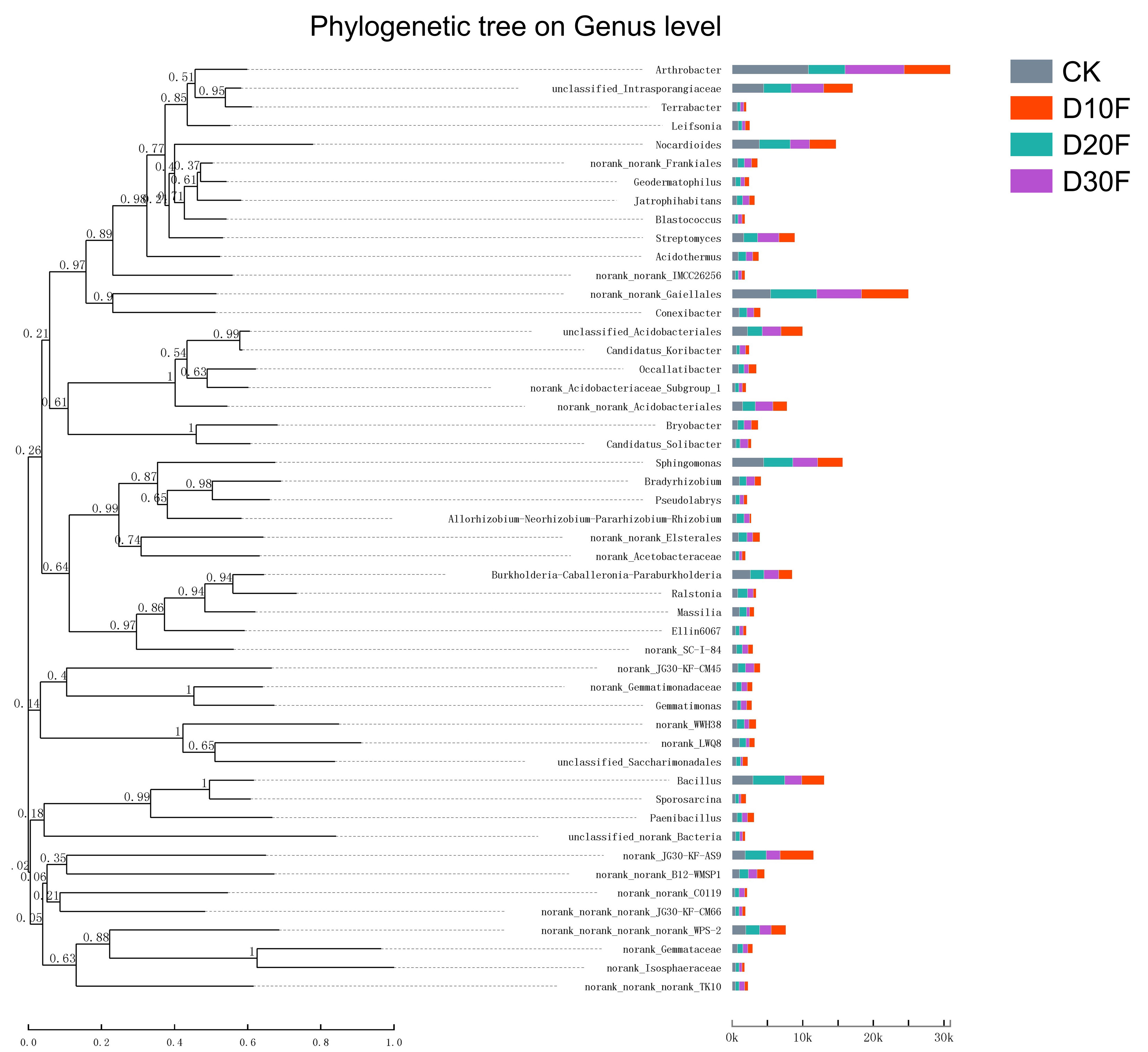
**

**Figure S1.** Composition and phylogenetic tree of bacterial communities of different treatments, based on genus level. The phylogenetic tree was calculated based on 16S rRNA sequencing alignments using the neighbor-joining method.

**Table S1. Correlation between top 50 genera, based on relative abundance.**

**Note:** As the Table S1 was too large to be placed in this Word, please check the Excel file named “Table S1: Correlation between top 50 genera, based on relative abundance”

**Table S2.** **The physicochemical data of the tobacco-planting soil.**

| **Treatments** | **WC/ %** | **pH** | **AK/ mg kg^-1^** | **AP/ mg kg^-1^** | **TN/ mg kg^-1^** | **SOM/ g kg^-1^** |
| --- | --- | --- | --- | --- | --- | --- |
| CK1 | 8.88 | 5.46 | 264.42 | 5.19 | 901.21 | 8.45 |
| CK2 | 8.88 | 5.43 | 265.86 | 5.00 | 877.29 | 8.34 |
| CK3 | 8.88 | 5.34 | 269.59 | 3.71 | 948.21 | 8.34 |
| D10F1 | 8.41 | 5.57 | 221.96 | 3.77 | 869.82 | 8.91 |
| D10F2 | 8.41 | 5.4 | 224.25 | 3.60 | 896.03 | 8.91 |
| D10F3 | 8.41 | 5.47 | 206.75 | 3.20 | 823.63 | 8.80 |
| D20F1 | 9.15 | 6.35 | 227.41 | 3.49 | 879.22 | 5.95 |
| D20F2 | 9.15 | 6.38 | 237.16 | 4.72 | 952.29 | 8.80 |
| D20F3 | 9.15 | 6.27 | 223.97 | 5.39 | 928.06 | 8.57 |
| D30F1 | 5.79 | 6.57 | 229.99 | 2.15 | 824.47 | 8.68 |
| D30F2 | 5.79 | 6.45 | 221.96 | 2.22 | 870.72 | 8.68 |
| D30F3 | 5.79 | 6.46 | 206.75 | 2.50 | 800.12 | 7.89 |

WC represented water content; AK represented available potassium; AP represented available phosphorus; TN represented total nitrogen; SOM represented soil organic matter.

**Table S3.** **The Enzyme data of the tobacco-planting soil.**

| **Treatments** | **S_SC** | **CAT** | **PPO** | **UE** | **ACP** | **NR** |
| --- | --- | --- | --- | --- | --- | --- |
| CK1 | 8.88 | 5.46 | 264.42 | 5.19 | 901.21 | 8.45 |
| CK2 | 8.88 | 5.43 | 265.86 | 5.00 | 877.29 | 8.34 |
| CK3 | 8.88 | 5.34 | 269.59 | 3.71 | 948.21 | 8.34 |
| D10F1 | 8.41 | 5.57 | 221.96 | 3.77 | 869.82 | 8.91 |
| D10F2 | 8.41 | 5.4 | 224.25 | 3.60 | 896.03 | 8.91 |
| D10F3 | 8.41 | 5.47 | 206.75 | 3.20 | 823.63 | 8.80 |
| D20F1 | 9.15 | 6.35 | 227.41 | 3.49 | 879.22 | 5.95 |
| D20F2 | 9.15 | 6.38 | 237.16 | 4.72 | 952.29 | 8.80 |
| D20F3 | 9.15 | 6.27 | 223.97 | 5.39 | 928.06 | 8.57 |
| D30F1 | 5.79 | 6.57 | 229.99 | 2.15 | 824.47 | 8.68 |
| D30F2 | 5.79 | 6.45 | 221.96 | 2.22 | 870.72 | 8.68 |
| D30F3 | 5.79 | 6.46 | 206.75 | 2.50 | 800.12 | 7.89 |

S_SC represented soil sucrase; CAT represented catalase; PPO represented polyphenol oxidase; UE represented urease; ACP represented acid phosphatase; NR represented nitrite reductase.
